# Identification of the Conserved and Novel miRNAs in Mulberry by High-Throughput Sequencing

**DOI:** 10.1371/journal.pone.0104409

**Published:** 2014-08-13

**Authors:** Ling Jia, Dayan Zhang, Xiwu Qi, Bi Ma, Zhonghuia Xiang, Ningjia He

**Affiliations:** State Key Laboratory of Silkworm Genome Biology, Southwest University, Beibei, Chongqing, China; New Mexico State University, United States of America

## Abstract

miRNAs are a class of non-coding endogenous small RNAs. They play vital roles in plant growth, development, and response to biotic and abiotic stress by negatively regulating genes. Mulberry trees are economically important species with multiple uses. However, to date, little is known about mulberry miRNAs and their target genes. In the present study, three small mulberry RNA libraries were constructed and sequenced using high-throughput sequencing technology. Results showed 85 conserved miRNAs belonging to 31 miRNA families and 262 novel miRNAs at 371 loci. Quantitative real-time PCR (qRT-PCR) analysis confirmed the expression pattern of 9 conserved and 5 novel miRNAs in leaves, bark, and male flowers. A total of 332 potential target genes were predicted to be associated with these 113 novel miRNAs. These results provide a basis for further understanding of mulberry miRNAs and the biological processes in which they are involved.

## Introduction

miRNAs, which are found in animals and plants, are a class of 19–24 nt non-coding small RNA molecules. They negatively regulate genes at the transcriptional and post-transcriptional level by cleaving the target mRNA and suppressing the translation of target mRNA [1]. miRNAs are encoded by *MIR* genes. In plants, *MIR* genes are transcribed into a pri-miRNA with a cap and a poly (A) tail and thereafter processed into a pre-miRNA, which is further cleaved into a miRNA/miRNA* duplex. The last nucleotide of the 3′ terminal in the duplex is methylated, and then the plant miRNA is loaded into the ARGONAUTE1 (AGO1) complex leading to the cleavage of the target mRNA [1]. Increasing amounts of evidence have demonstrated that miRNAs play crucial roles in plant growth, development, and response to biotic and abiotic stress [2]–[4]. For example, miR156 regulates the transition of juvenile to adult in *Arabidopsis thaliana* [5]. miR172 negatively regulates the cell fate specification in flower development of *A. thaliana* [6]. miR160 controls the formation of root caps by targeting auxin response factors ARF10 and ARF16, both of which restrict the stem cell niche and promote columella cell differentiation [7].

Despite the importance of miRNA, the first miRNA, lin-4, was discovered until 1993 from *Caenorhabditis elegans* [8]. In 2001, tens of miRNA were identified in several animal species by directly cloning and sequencing [9], [10]. Since then, bioinformatic prediction and cloning have been used to identify many miRNAs in animals and plants [11]–[15]. However, although predictions that rely on the sequence can predict the conserved miRNAs easily, it difficult to identify species-specific miRNAs. The cloning method can only be used to identify small-scale miRNAs. In 2005, high-throughput sequencing technology was first used to sequence the small RNA libraries of *A. thaliana* and many miRNAs were identified [16]. This next-generation sequencing technology enables massive sequencing and detection of minimally abundant small RNA. It has become technique of choice for sequencing of the genome, transcriptome, and small RNA transcriptome [17]–[21]. A recent analysis of miRNA (based on Release 19, http://www.mirbase.org/, August 2012) showed a total of 21,264 miRNAs to be registered in miRBASE. This number is almost 20 times than that in Release 6.0 (Release 6.0, ftp://mirbase.org/pub/mirbase/CURRENT/README, April 2005). The expansion of currently available miRNA information can be attributed to the development of this technique.

Mulberry trees are widely planted in Europe, Africa, Asia, and the United States. This tree belongs to the genus *Morus*, family Moraceas, order Rosales [22], [23]. Mulberry leaves have been used to feed silkworms for silk production for about 5,000 years. In addition to being the sole nutritional source of the silkworm, mulberry tree have many other multiple uses. In particular, the secondary metabolites of mulberry plants are widely used as medicines [24]–[26]. In the current genomic era, the genome data are an important resource for gene identification and characterization. The completion of the mulberry genome has allowed scholars to look at mulberry genes comprehensively [18]. Deep sequencing of transcripts can reveal many important gene products, such as miRNAs, which have been shown to be crucial to plant development and stress responses [2]–[4]. For this reason, mulberry miRNAs were sequenced and analyzed in the present study. Three small RNA libraries of mulberry tissues (leaves, bark, and male flowers) were constructed and used for sequencing. Conserved, novel mulberry's miRNAs and their target genes were identified. The expression profiles of 9 conserved and 5 novel mulberry miRNAs were confirmed in three tissues using stem-loop quantitative real-time PCR. These results expand our knowledge of the diversity and specificity of mulberry miRNAs and provide a basis for further understanding of the biological mechanisms that take place in mulberry plants.

## Materials and Methods

### Plant material and construction of small RNA library

The wild mulberry species *Morus notabilis* grows in a pristine forest in Yaan, Sichuan Province, in southwest of China. This tree is located at 29°45.278' north latitude, 102°53.878' east longitude. It is a mulberry species used for the genome sequencing. Young leaves, bark and male flowers from *M. notabilis* were collected in the spring of 2013. The samples were immediately frozen in liquid nitrogen and stored in −80°C. No specific permissions were required for these activities. The field studies did not involve endangered or protected species. Small RNA libraries of *M. notabilis* were constructed as described elsewhere with a few modifications [27]. Briefly, the total RNA of three *M. notabilis* tissues was extracted using RNAiso plus (D9108A, Takara, China) in accordance with the manufacturers' instruction. Samples were then subjected to 15% denaturing polyacrylamide gel electrophoresis (PAGE). Small RNA fragments of 18–30 nt were separated, purified, and ligated with the 5′, 3′adapter sequentially. After reverse transcription and PCR, about 20 µg products of three tissues were separately sequenced using Illumina HiSeq-2000 (BGI-Shenzhen, China).

### Bioinformatics analysis of small RNA

Raw data were filtered using a Perl script to delete low-quality reads, chip adapter sequences, and contaminations. The sequences ≥18 nt of clean data were annotated in the Rfam database (Release 10.1) (http://www.sanger.ac.uk/software/Rfam) and Genbank non-coding RNA database (http://www.ncbi.nlm.nih.gov/) to remove non-coding RNA (rRNA, tRNA, snRNA, snoRNA) and degradation fragments of mRNA. The remaining sequences were aligned against miRNA database, miRBASE (Release 19) (http://www.mirbase.org/), and perfectly matched sequences were considered conserved *M. notabilis* miRNAs.

### Prediction of novel miRNAs

The unannotated small RNAs 18–30 nt in length were searched against *M. notabilis* genome. Novel *M. notabilis* miRNA were predicted using Mireap software (http://sourceforge.net/projects/mireap/) with modifications basing on the default parameters: (1) the miRNA sequence length was 18–25 nt; (2) the miRNA reference sequence length was 20–23 nt; (3) the maximum copy number of miRNAs in any of the previous studies was 20; (4) the free energy of miRNA precursor was less than −18 kcal/mol; (5) the maximum space between miRNA and miRNA* was 300 nt; (6) the minimal base pairs of miRNA and miRNA* was 16; (7) the maximum bulge of miRNA and miRNA* was 4; (8) the maximum asymmetry of miRNA/miRNA* duplex was 4; and (9) the flank sequence length of miRNA precursor was 20 nt. Hairpin structures of potential novel miRNA precursors were checked manually. The criteria were as follows: (1) the minimal folding free energy index (MFEI) of potential novel miRNA precursor was required be at least 0.85 [28]; (2) the asymmetric bulges of miRNA/miRNA* duplex were less than 3; (3) there were fewer than 4 mismatches between miRNA and miRNA*; (4) if the miRNA sequence did not fit these criteria, but corresponding miRNA* were detected, miRNA were also considered potentially novel miRNA.

### Expression of mulberry miRNAs and target genes as assessed using qRT-PCR

Fourteen miRNAs were chosen for stem-loop RT-PCR in three mulberry tissues as previous described [29]. Briefly, each 1 µg total RNA was hybridized with a miRNA-specific stem-loop primer (10 pmol). The hybridized miRNA molecules were reverse transcribed to cDNA in 10 µL reaction using Reverse Transcriptase M-MLV (2641A, TaKaRa, China) in accordance with the manufacturers' instruction. The resultant was then diluted three-fold and 1.5 µL cDNA was used as the template to perform the stem-loop RT-PCR with each miRNA specific forward primer and universal primer, as listed in Table S7. The reverse transcriptions for target genes were performed as follows. One microgram of total RNAs were reverse transcripted a 20 µL reaction using PrimerScript RT Reagent kit with gDNA Eraser (RR047A, TaKaRa, China) basing on the handbook described. The resultant was then diluted four-fold and 1 µL cDNA was used as the template to perform the RT-PCR with each target gene primers, as listed in Table S8. The PCR reactions were performed in ABI Step One Plus (Applied Biosystems, USA) using SYBR Premix Ex Taq II (RR820A, TakaRa, China) as the following conditions: 95°C for 30 s, 40 cycles of 95°C for 5 s and 60°C for 30 s. The 5.8S rRNA and ribosomal protein L15 gene were used as inner controls. All reactions were assayed in triplicated. The relative expression level of miRNA was calculated using 2^−ΔΔCt^ method.

### Prediction of miRNA targets

miRNA target prediction was performed by aligning miRNAs with the *M. notabilis* genes using a perl script, which was designed to predict the miRNA targets according to the criteria described by Allen and Schwab [30] [31]. These criteria were as follows: (1) There had to be no more than 4 mismatches in the miRNA/target duplex (G–U pairs were considered 0.5 of a mismatch). (2) There had to be no more than 2 adjacent mismatches in the miRNA/target duplex. (3) There had to be no adjacent mismatches at position 2–12 at the miRNA 5′ terminal of the miRNA/target duplex. (4) There had to be no mismatches in the position 10 and 11 of the miRNA/target duplex. (5) There had to be no more than 2.5 mismatches in the position of 1–12 during the miRNA/target duplex. (6) The minimal free energy of miRNA/target duplex had to exceed 75% that of the miRNA when bound to its perfect complement.

## Results

### Small RNA in three mulberry tissues

In order to identify the miRNAs in mulberry plants, total RNA (integrity ≥8.0) was extracted from three mulberry tissues and three small RNA libraries were constructed for sequencing. These data have been deposited in NCBI/SRA database under accession number of SRP032829.

**Figure 1:**
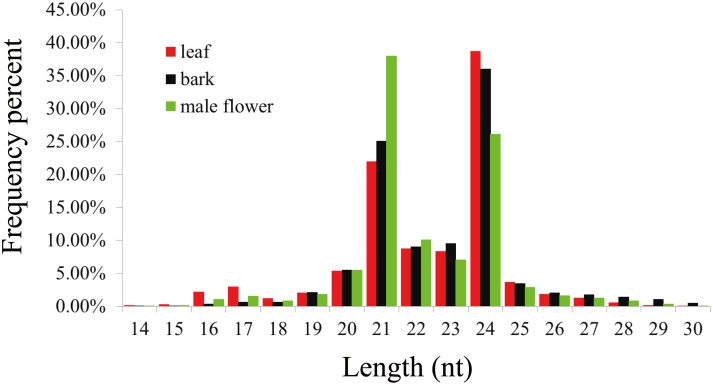
Length distribution of small RNAs from mulberry leaves, bark, and male flowers. The X axis represents the length of small RNAs. The Y axis represents the relative frequency.

A total of 11,752,747 reads (leaf), 11,491,921 reads (bark), and 10,513,612 reads (male flower) were obtained by sequencing, as shown in Table 1. After removing low quality sequences, adapters, and contaminated sequences, there were 10,992,174 (93.97%) clean reads ≥18 nt in size from leaves, 11,273,911 (98.55%) from bark, and 10,134,148 (96.83%) from male flowers. Among the clean reads ≥18 nt in size, the reads of miRNAs was 832,571 (leaf), 1,130,016 (bark), 2,359,403 (male flower). The total reads of three tissues were subjected to analyze the size distribution as shown in Figure 1. The 21–24 nt small RNAs made up 77.87%, 79.78%, and 81.39% of the reads from mulberry leaves, bark, and male flowers accounted, respectively. The most common size of small RNAs in leaves and bark was 24 nt, accounting for 38.75% and 36.04% of the total, respectively. This was consistent with the size distribution patterns of small RNAs in *Arachis hypogaea* and *Raphanus sativus* [32], [33]. However, the size distribution pattern of male mulberry flowers was different. The most common size for small RNAs in male flower tissue was 21 nt, which was consistent with *Fragaria × ananassa* and *Pinus contorta* [34], [35].

**Table 1:** Classification of small RNAs in the mulberry leaves, bark, and male flowers.

Category	Leaves		Bark		Male flowers	
	unique reads	total reads	unique reads	total reads	unique reads	total reads
Raw data		11,752,747		11,491,921		10,513,612
high quality		11,697,570		11,439,285		10,465,855
clean_reads [size > = 18 nt]	3,383,828	10,992,174	3,848,791	11,273,911	2,851,747	10,134,148
match genome build2	2,619,858	9,394,886	2,776,320	9,122,054	2,207,022	8,601,051
exon_antisense	75,103	195,404	77,783	178,514	74,619	160,433
exon_sense	175,011	401,988	151,119	308,470	150,431	306,205
intron_antisense	140,785	298,027	153,833	314,352	108,403	196,686
intron_sense	222,666	865,955	236,136	714,362	179,397	511,639
miRNAs	26,956	832,571	31,463	1,130,016	25,805	2,359,403
rRNA	114,561	2,157,926	162,589	1,886,493	111,055	1,885,168
repeat	250,148	525,805	223,690	414,606	263,197	435,246
snRNA	2,882	13,281	4,210	17,357	3,754	18,088
snoRNA	1,058	3,169	1,148	2,900	831	1,816
tRNA	12,054	368,395	20,001	378,792	13,571	335,167
unannotated small RNA	2,362,604	5,329,653	2,786,819	5,928,049	1,920,684	3,924,297

### Conserved miRNAs in mulberry plants

In order to investigate the conserved miRNAs in mulberry plants, unique small RNAs from three mulberry tissues were aligned against miRNAs registered in the miRBase database (Release 19). Using the principle of sequence perfect matching, the present analysis identified 85 conserved miRNAs belonging to 31 families, as shown in Table 2. There were 77, 70, and 70 miRNAs identified in leaf, bark, and male flower tissue, respectively. Among the 85 conserved miRNAs, 57 were common to all three tissue libraries (Figure S1B). The length distribution of 85 conserved miRNAs is shown in Figure S1A. A peak appeared at 21 nt (84.7%).

**Table 2:** Conserved miRNAs in three types of mulberry tissues.

Family	Name	Reference miRNA	Sequence (5'-3')	Length	Reads in leaf	Reads in bark	Reads in male flower	*Ath*	*Gma*	*Mdm*	*Ptc*	*Rco*	*Osa*	*Zma*
miR156	mno-miR156a	zma-miR156g-3p	GCTCACTTCTCTTTCTGTCAGC	22	0	46	4	-	-	-	-	-	++	++
	mno-miR156b	gma-miR156f	TTGACAGAAGAGAGAGAGCACA	22	6	2	337	+	++	+	+	+	+	+
	mno-miR156c	gma-miR156m	TTGACAGAAGATAGAGAGCAC	21	12,787	117,270	768,488	++	++	++	++	+	+	+
	mno-miR156d	gma-miR156o	TTGACAGAAGAGAGTGAGCAC	21	2,942	14,574	8,723	-	++	+	+	+	-	+
	mno-miR156e	mdm-miR156w	TTGACAGAAGAGAGAGAGCAC	21	2,099	1,277	155,199	+	++	++	+	+	+	+
	mno-miR156f	ssl-miR156	TGACAGAAGAGAGTGAGCACA	21	22	378	65	-	+	+	+	++	-	+
	mno-miR156g	tcc-miR156a	TGACAGAAGAGAGAGAGCACA	21	8	4	1,198	+	++	+	-	++	++	++
miR159	mno-miR159a	ssp-miR159a	TTTGGATTGAAGGGAGCTCTG	21	3,624	4,571	3,146	+	+	+	+	+	++	++
	mno-miR159b	aly-miR159c-3p	TTTGGATTGAAGGGAGCTCCT	21	31	29	0	++	+	+	+	+	+	+
miR160	mno-miR160a	aly-miR160a-3p	GCGTATGAGGAGCCATGCATA	21	596	428	2,345	-	+	-	++	-	-	-
	mno-miR160b	cme-miR160c	TGCCTGGCTCCCTGTATGCCA	21	11	14	13	++	++	++	++	++	++	++
miR162	mno-miR162	cme-miR162	TCGATAAACCTCTGCATCCAG	21	578	956	727	++	++	++	++	++	++	+
miR164	mno-miR164a	cme-miR164d	TGGAGAAGCAGGGCACGTGCA	21	10,734	6,078	1,191	++	++	++	++	++	++	++
	mno-miR164b	vun-miR164	TGGAGAAGGGGAGCACGTGCA	21	21	0	12	+	+	+	+	+	+	+
miR166	mno-miR166a	hbr-miR166b	TCGGACCAGGCTTCATTCCCCC	22	26	51	27	-	+	+	+	+	-	-
	mno-miR166b	cme-miR166a	TCGGACCAGGCTTCATTCCCC	21	198,685	238,486	252,806	-	++	++	++	++	-	-
	mno-miR166c	gma-miR166k	TCTCGGACCAGGCTTCATTCC	21	20,100	168,078	39,124	-	++	+	-	+	-	-
	mno-miR166d	gma-miR166l	GGAATGTTGTCTGGCTCGAGG	21	3,935	975	901	-	++	-	-	-	++	++
	mno-miR166e	osa-miR166g-3p	TCGGACCAGGCTTCATTCCTC	21	91	241	121	-	+	+	+	+	++	++
	mno-miR166f	bdi-miR166e	CTCGGACCAGGCTTCATTCCC	21	31	56	194	-	+	+	-	+	-	-
	mno-miR166g	zma-miR166m-5p	GGAATGTTGGCTGGCTCGAGG	21	23	0	4	-	+	-	-	-	++	++
	mno-miR167h	mdm-miR167h	TGAAGCTGCCAGCATGATCTTA	22	1,410	18	881	-	+	++	+	+	-	+
miR167	mno-miR167b	bna-miR167b	TGAAGCTGCCAGCATGATCTAA	22	574	84	1,526	+	+	+	+	+	+	+
	mno-miR167c	nta-miR167c	TGAAGCTGCCAGCATGATCTGG	22	115	125	28	++	+	+	+	++	+	+
	mno-miR167a	ath-miR167a	TGAAGCTGCCAGCATGATCTA	21	75,303	12,129	140,989	++	++	++	++	++	++	++
	mno-miR167d	cme-miR167c	TGAAGCTGCCAGCATGATCTT	21	3,722	324	4,334	-	++	++	++	+	-	+
	mno-miR167e	cme-miR167f	TGAAGCTGCCAGCATGATCTG	21	47	21	43	++	++	+	++	++	++	++
miR168	mno-miR168a	aau-miR168	GATCCCGCCTTGCATCAACTGAAT	24	9	27	12	-	-	-	+	-	+	-
	mno-miR168b	mdm-miR168b	TCGCTTGGTGCAGGTCGGGAA	21	18,898	33,236	31,342	++	++	++	++	++	+	+
	mno-miR168c	mtr-miR168c-3p	CCCGCCTTGCATCAACTGAAT	21	246	891	476	-	-	-	++	-	-	+
miR169	mno-miR169a	cme-miR169f	CAGCCAAGGATGACTTGCCGG	21	113	386	1,314	++	++	++	++	++	++	++
	mno-miR169c	nta-miR169p	CAGCCAAGGATGACTTGCCGA	21	73	0	106	++	++	+	++	+	++	++
	mno-miR169b	zma-miR169b-3p	GGCAAGTTGTTCTTGGCTACA	21	1	0	0	-	+	-	+	-	-	++
miR171	mno-miR171a	cme-miR171f	TGATTGAGCCGTGCCAATATC	21	379	5	528	+	++	++	++	++	++	++
	mno-miR171d	mdm-miR171l	TTGAGCCGCGCCAATATCACT	21	36	9	3	+	++	++	+	+	+	+
	mno-miR171b	gma-miR171b-3p	CGAGCCGAATCAATATCACTC	21	34	603	127	-	++	+	+	+	+	+
	mno-miR171e	mdm-miR171n	TTGAGCCGTGCCAATATCACA	21	32	1	0	+	++	++	+	+	-	++
	mno-miR171c	ptc-miR171c	AGATTGAGCCGCGCCAATATC	21	29	75	27	+	+	+	++	++	+	+
	mno-miR171f	gma-miR171j-5p	TATTGGCCTGGTTCACTCAGA	21	6	0	2	-	++	-	-	-	+	+
	mno-miR171g	mdm-miR171b	TTGAGCCGCGTCAATATCTCC	21	5	600	8	-	+	++	-	-	+	+
	mno-miR171h	cme-miR171b	TTGAGCCGTGCCAATATCACG	21	4	0	5	++	++	++	++	++	-	+
	mno-miR171i	gma-miR171l	CGATGTTGGTGAGGTTCAATC	21	2	0	0	-	++	-	-	-	+	-
miR172	mno-miR172a	cme-miR172c	AGAATCTTGATGATGCTGCAT	21	19,265	8,484	1,245	++	++	++	++	-	++	+
	mno-miR172b	cme-miR172e	AGAATCTTGATGATGCTGCAG	21	143	13	137	++	+	++	+	+	+	+
	mno-miR172c	cme-miR172d	GGAATCTTGATGATGCTGCAT	21	15	266	6	++	++	++	++	+	++	++
	mno-miR172d	aly-miR172c-5p	GGAGCATCATCAAGATTCACA	21	11	6	4	-	+	-	++	-	+	+
	mno-miR172e	tcc-miR172d	AGAATCCTGATGATGCTGCAT	21	3	2	0	+	+	+	+	-	+	+
	mno-miR172f	mtr-miR172c-5p	GTAGCATCATCAAGATTCACA	21	1	6	0	-	+	-	+	-	+	+
miR319	mno-miR319a	tcc-miR319	TTTGGACTGAAGGGAGCTCCT	21	2	0	9	+	+	+	+	+	+	+
	mno-miR319b	mdm-miR319b	TTGGACTGAAGGGAGCTCCCT	21	0	57	0	++	++	++	+	++	+	+
	mno-miR319c	ppt-miR319e	CTTGGACTGAAGGGAGCTCCC	21	0	4	0	+	+	+	+	+	+	+
miR390	mno-miR390	cme-miR390c	AAGCTCAGGAGGGATAGCGCC	21	4,275	489	1,153	++	++	++	++	++	++	++
miR393	mno-miR393a	aly-miR393a-3p	ATCATGCTATCTCTTTGGATT	21	36	25	37	-	-	-	+	-	-	-
	mno-miR393b	cme-miR393c	TCCAAAGGGATCGCATTGATC	21	2	4	2	++	++	++	++	++	++	++
	mno-miR393c	mdm-miR393c	TCCAAAGGGATCGCATTGATCT	22	0	103	19	+	+	++	+	+	++	++
miR395	mno-miR395a	cca-miR395c	CTGAAGTGTTTGGAGGAACTC	21	0	2	0	+	+	+	+	+	+	+
	mno-miR395b	cme-miR395f	CTGAAGTGTTTGGGGGAACTC	21	43	104	47	++	++	++	++	++	+	+
miR396	mno-miR396a	gma-miR396k	GCTCAAGAAAGCTGTGGGAGA	21	848	149	135	-	++	-	+	-	+	+
	mno-miR396b	cme-miR396b	TTCCACAGCTTTCTTGAACTG	21	190	2,750	657	++	++	++	++	+	++	++
	mno-miR396c	cme-miR396d	TTCCACAGCTTTCTTGAACTT	21	181	57	105	++	++	++	++	++	++	++
	mno-miR396d	gma-miR396i-3p	GTTCAATAAAGCTGTGGGAAG	21	12	128	47	-	++	-	-	-	+	+
miR397	mno-miR397a	cme-miR397	TCATTGAGTGCAGCGTTGATG	21	211	154	1,483	++	++	+	++	++	++	+
	mno-miR397b	osa-miR397b	TTATTGAGTGCAGCGTTGATG	21	0	1	0	+	+	+	+	+	++	+
miR398	mno-miR398	cme-miR398a	TGTGTTCTCAGGTCGCCCCTG	21	130	32	332	+	++	++	++	++	++	+
miR399	mno-miR399a	mdm-miR399c	TGCCAAAGGAGAATTGCCCTG	21	179	3	0	+	+	++	++	+	++	++
	mno-miR399b	mdm-miR399j	TGCCAAAGGAGAGTTGCCCTG	21	111	1	83	++	++	++	+	++	++	++
	mno-miR399c	zma-miR399e-5p	GGGCTTCTCTTTCTTGGCAGG	21	36	0	16	-	-	-	-	-	-	++
	mno-miR399d	gma-miR399g	TGCCAAAGGAGATTTGCCCAG	21	26	4	71	+	++	+	+	++	++	+
	mno-miR399e	cme-miR399a	TGCCAAAGGAGATTTGCCCCG	21	11	0	0	++	+	+	++	+	+	+
	mno-miR399f	cme-miR399c	TGCCAAAGGAGATTTGCCCGG	21	9	2	28	++	+	+	++	++	+	+
miR408	mno-miR408a	nta-miR408	TGCACTGCCTCTTCCCTGGCT	21	0	0	2	+	+	+	+	+	+	+
	mno-miR408b	smo-miR408	TGCACTGCCTCTTCCCTGGCTG	22	2	0	6	+	+	+	+	+	+	+
	mno-miR408c	cme-miR408	ATGCACTGCCTCTTCCCTGGC	21	219	59	838	++	++	++	++	+	+	+
miR482	mno-miR482	mdm-miR482a-5p	AGGAATGGGCTGTTTGGGAAGA	22	23	54	25	-	-	++	-	-	-	-
miR529	mno-miR529a	osa-miR529b	AGAAGAGAGAGAGTACAGCTT	21	2,851	367	6,790	-	-	-	-	-	++	+
	mno-miR529b	far-miR529	AGAAGAGAGAGAGCACAGCTT	21	3	0	5	-	-	-	-	-	+	+
miR535	mno-miR535	mdm-miR535a	TGACAACGAGAGAGAGCACGC	21	21,566	57,272	19,329	-	-	++	-	++	++	-
miR827	mno-miR827	mdm-miR827	TTAGATGACCATCAACGAACA	21	2	0	0	+	-	++	+	-	+	+
miR828	mno-miR828	cme-miR828	TCTTGCTCAAATGAGTATTCCA	22	5	1	0	+	++	++	++	-	-	-
miR858	mno-miR858	ath-miR858b	TTCGTTGTCTGTTCGACCTTG	21	17	17	2	++	-	+	-	-	-	-
miR2111	mno-miR2111	cme-miR2111b	TAATCTGCATCCTGAGGTTTA	21	11	10	10	++	++	++	+	-	-	-
miR4376	mno-miR4376	gma-miR4376-5p	TACGCAGGAGAGATGACGCTGT	22	9,535	1,231	6,682	-	++	+	-	-	-	-
miR4414	mno-miR4414	mtr-miR4414a-5p	AGCTGCTGACTCGTTGGTTCA	21	121	92	6	-	+	-	-	-	-	-
miR4995	mno-miR4995	gma-miR4995	AGGCAGTGGCTTGGTTAAGGG	21	10	0	0	-	++	-	-	-	-	-
miR5523	mno-miR5523	osa-miR5523	TGAGGAGGAACATATTTACTAG	22	0	1	2	-	-	-	-	-	++	-

"++" represents the mulberry miRNAs that are perfectly matched to those of other plant species. "+" represents mulberry miRNAs with 1–3 mismatches relative to the seven other plant miRNAs. "-" represents mulberry miRNAs with more than 3 mismatches relative to those of seven other plant species. *ath*, *gma*, *mdm*, *ptc*, *rco*, *osa*, *zma* and *mno* indicate *Arabidopsis thaliana*, *Glycine max*, *Malus domestica*, *Populus trichocarpa*, *Ricinus communis*, *Oryza sativa*, *Zea mays* and *Morus notabilis*, respectively.

In a broader evolutionary context, mulberry miRNAs were compared to those of seven other plants, including five dicotyledons (*A. thaliana*, *Glycine max*, *Malus domestica*, *Populus trichocarpa*, *Ricinus communis*) and two monocotyledons (*Oryza sativa* and *Zea mays*). Of the 31 mulberry miRNA families, 24 were conserved in the seven plant species. These miRNA were classified into well-conserved miRNA families. Prominent among them were mulberry miR160b, miR164a, miR167a, miR169a, miR390, and miR396b, which completely matched their counterparties in the seven other plant species, suggesting those miRNAs were extremely conserved, and might play critical physiological roles in both dicotyledons and monocotyledons. However, 7 miRNA families, miR482, miR529, miR858, miR4376, miR4414, miR4995, and miR5523, were found in only one or two plant species (Table 2).

It has been reported that the sequencing frequency in Illumina technology was used to estimate the relative levels of expression of miRNAs [32]. The present data indicated that the conserved miRNA families were expressed across a vast range, from over 10,000 reads to fewer than 10 reads in mulberry, as shown in Table 2. Of the 31 miRNA families, the reads of miR156, miR166, miR167, miR168, and miR535 exceeded 10,000 in all three tissues. Eleven miRNA families (miR159, miR160, miR164, miR169, miR171, miR172, miR390, miR396, miR397, miR529 and miR4376) had more than 1000 reads at least in one tissue. Seven miRNA families (miR162, miR393, miR395, miR398, miR399, miR408 and miR4414) had numbers of sequence reads ranging from 100–1000 at least in one tissue, and the remaining miRNA families (miR319, miR482, miR827, miR828, miR858, miR2111, miR4995 and miR5523) had fewer than 100 reads in all three tissues.

### Novel miRNAs in mulberry plants

One of the greatest advantages of high-throughput sequencing is that this technology can be used to discover species-specific miRNAs. Here, mireap software was used with several criteria to identify the novel mulberry miRNAs, as described in method section. Using un-annotated sequences from mulberry three tissues, 262 novel miRNAs in 371 mulberry genome loci were identified, as shown in Table S1. Previous studies have reported that miRNA* sequence can be used as criteria for the identification of novel miRNAs. In the present study, 90 miRNA* sequences were discovered in the precursors of novel miRNAs. This suggested that those 90 novel miRNAs must be real mulberry miRNAs. Most of the 90 miRNAs* had much lower abundance than their partial strand miRNAs. However, several miRNAs*(mno-miRn166*, mno-miRn76a-2*, and mno-miRn82*) exhibited almost the same abundance with their partners in male flowers. Specifically, mno-miRn82* had higher abundance than its miRNA in leaf tissue. The 262 novel mature miRNAs and 371 miRNA precursors were considered together, and the lengths of the miRNA precursors ranged from 65–357 nt with an average size of 142 nt. The majority were 65–189 nt in length, accounting for 87% of novel miRNA precursors. This was similar to those in *A. hypogae*a [32]. The minimal folding energy of the miRNA precursors varied from −225.7∼−21 kcal/mol with an average value of −56.50 kcal/mol, which was higher than that in tRNA (−27.5 kcal/mol) and rRNA (−33 kcal/mol) [36]. The majority of novel mulberry miRNAs identified in the present study were expressed at low levels with fewer than 100 reads.

### Identification of tissue-biased miRNAs

**Figure 2:**
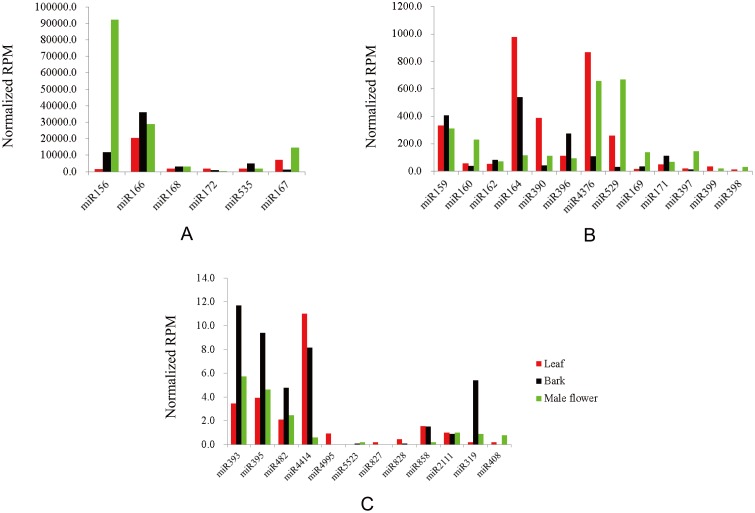
Reads per million of conserved miRNAs in mulberry leaves, bark, and male flowers. A) miRNAs with >1000 reads; B) miRNAs with 30–1000 reads; C) miRNAs with <30 reads. The X axis represents different conserved miRNAs. The Y axis represents the reads per million for different miRNAs. RPM means reads per million.

Some miRNAs were expressed in a tissue-specific/biased manner. Documentation of such miRNAs has served as foundational information for functional studies. In the present study, tissue-biased miRNA families were investigated by normalizing the reads in different data sets. miRNAs were considered tissue-biased if there were twice as many normalized reads in one tissue than in the other two tissues. As shown in Figure 2 and Table S2, 5 leaf-biased miRNA families (miR4995, miR827, miR828, miR172, and miR390), 5 bark-biased miRNA families (miR535, miR396, miR393, miR395, and miR319), and 8 male flower-biased miRNA families (miR156, miR529, miR160, miR397, miR398, miR408, miR169, and miR167) were identified in conserved mulberry miRNA families. Among mulberry novel miRNAs, 58 leaf-biased, 84 bark-biased, and 72 male flower-biased miRNAs were identified, as shown in Table S3. Among these tissue-biased novel miRNAs, 8, 6, and 15 novel miRNAs were only observed in leaf, bark, and male flower tissue, respectively (Table S4). These 29 novel miRNAs had very few reads, ranging in number from 5–136.

### Detection of the expression of miRNAs using RT-PCR

**Figure 3:**
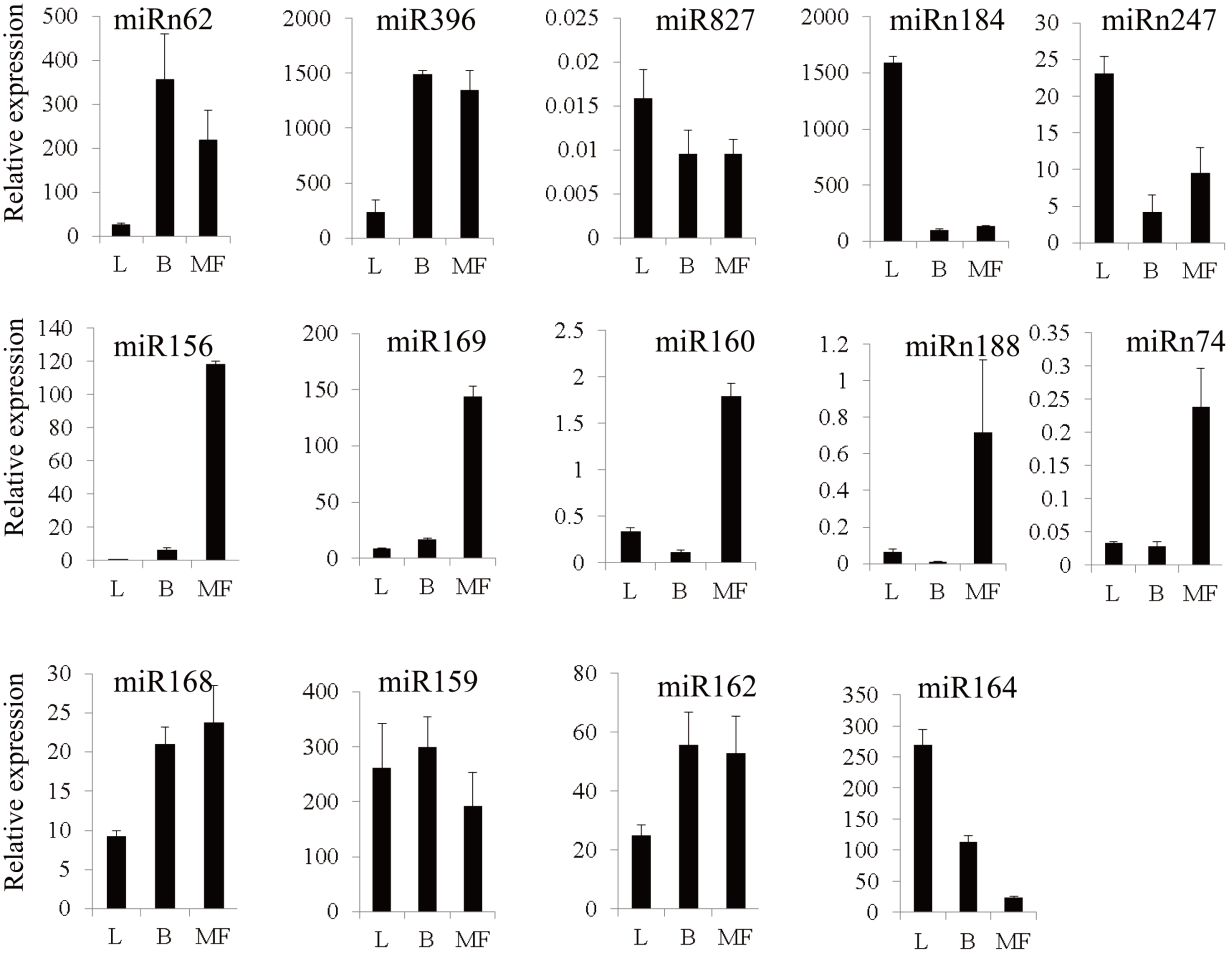
Relative expression of 14 miRNAs in three mulberry tissues (leaf, bark, and male flower) using stem-loop RT-PCR. The X axis represents different tissues. L indicates leaf tissue. B indicates bark tissue. MF indicates male flower tissue. The Y axis represents the relative expression level of miRNAs.

To confirm the expression pattern of the mulberry miRNAs, 9 conserved and 5 novel miRNAs with different expression profiles were randomly selected for stem-loop RT-PCR analysis. As illustrated in Figure 3, miR827, miRn247, and miRn184 were more abundantly expressed in leaves than in bark or male flowers. miR396 and miRn62 were highly expressed in bark. Five mulberry miRNAs, miR156, miR160, miR169, miRn74, and miRn188, were expressed predominantly in male flowers. The remaining 4 non-tissue-biased conserved miRNAs (miR159, miR162, miR164, and miR168) exhibited expression pattern nearly identical to the results of the analysis of sequencing data. Taken together, the results of stem-loop RT-PCR were consistent with the expression pattern of the tissue-biased miRNAs identified using high-throughput sequencing.

### Prediction of miRNA targets

Plant miRNAs play important roles in biological processes by cleaving target mRNAs and suppressing the translation of target genes. In order to understand the biological functions of mulberry miRNAs, the target genes of 31 mulberry conserved miRNAs representing 31 families with high reads and 262 novel miRNAs were predicted using the methods described above. As listed in Table S5 and Table S6, 89 target genes for 20 conserved miRNAs and 332 target genes for 113 novel miRNAs were annotated using the nr database. The majority of the target genes of conserved miRNAs were transcriptional factors, and as many miRNA targets were found to be conserved in mulberry plants as other plant species. These include miRNA-target pair associated with flower development, miR156-squamosa promoter-binding-like protein (SPL), miR159-MYB, miR166-homeobox-leucine zipper protein (HD-ZIP III), miR172-floral homeotic protein APETALA and pairs associated with root development, miR164-NAC domain-containing protein and miR167-auxin response factor 6 (ARF). Functional proteins were also identified as targets of conserved miRNA including mno-miR397 (laccase), mno-miR395 (sulfate transporter), and mno-miR390 (receptor-like protein kinase). The targets of novel miRNAs were mainly associated with protein-coding genes. For example, flavonol synthase/flavanone 3-hydroxylase, isoflavone 2'-hydroxylas, polyphenoloxidase, disease resistance protein, E3 ubiquitin-protein ligase, basic proline-rich protein, aspartyl-tRNA synthetase, anthocyanidin 5,3-O-glucosyltransferase, abscisic insensitive 1B, chitinase-like protein and cysteine-rich receptor-like protein kinase.

### Detection of the expression of target genes using RT-PCR

**Figure 4:**
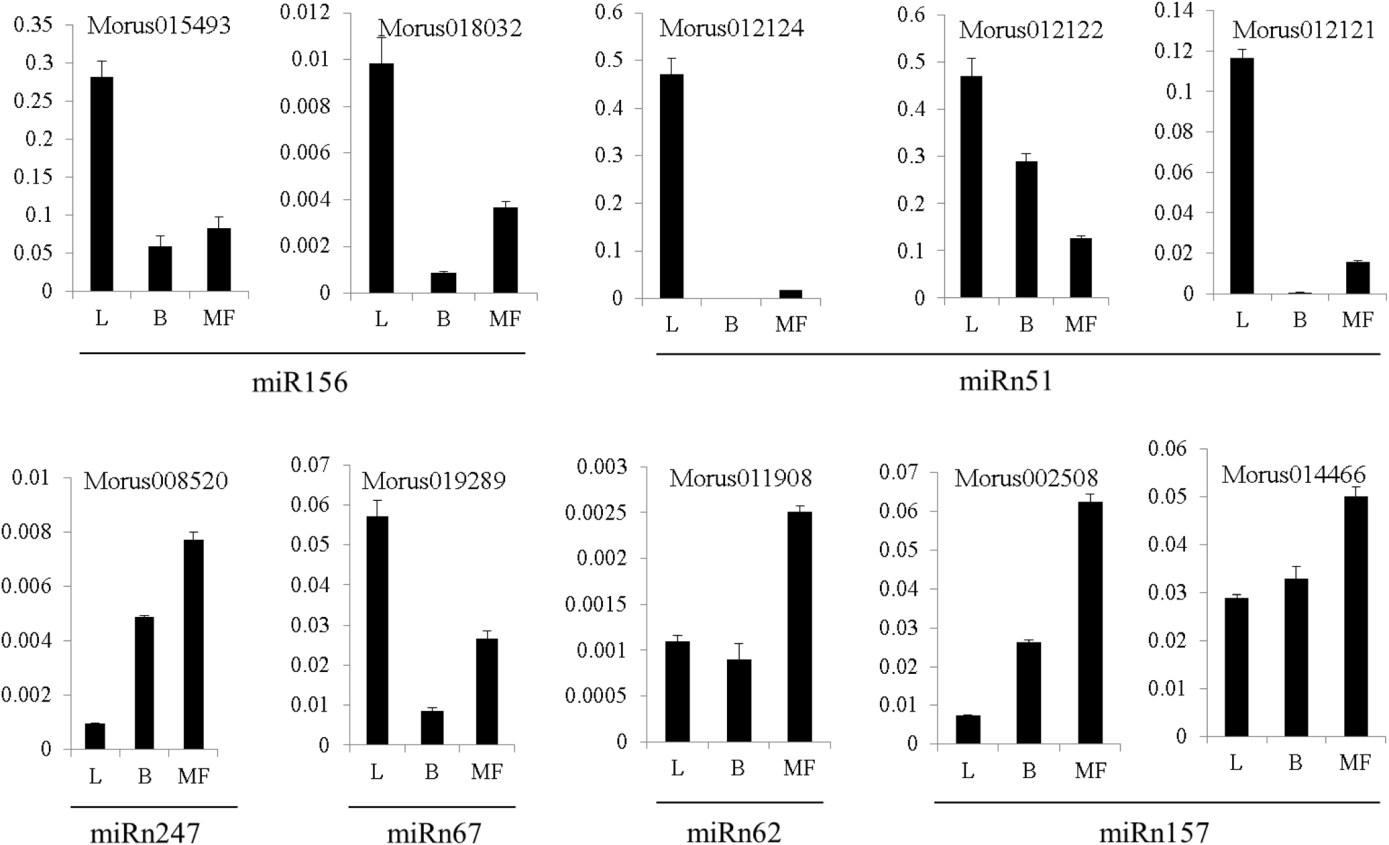
The expression profile of 10 target genes belonging to 6 miRNAs in three mulberry tissues (leaf, bark, and male flower) using RT-PCR. Morus015493 and Morus018032 are the predicted target genes of miR156, so as Morus012124, Morus012122, and Morus012121 for miRn51, Morus008520 for miRn247, Morus019289 for miRn67, Morus011908 for miRn62, Morus002508 and Morus014466 for miRn157. The X axis represents different tissues. L indicates leaf tissue. B indicates bark tissue. MF indicates male flower tissue. The Y axis represents the relative expression level of target genes. MorusXXXXXX represented the target gene ID.

miRNAs would decrease the mRNA or protein level of their regulating target genes [1]. To explore whether the potential predicted miRNA targets could be regulated by miRNAs, the expression profiles of 10 target genes for one conserved miRNA and 5 novel miRNAs were investigated. As illustrated in Figure 3 and Figure 4, the predicted target genes *Morus015493* and *Morus018032* of miR156 were lowly expressed in male flower and highly expressed in leaf, which was opposite to the expression pattern of miR156. In addition, as shown in Figure 4 and Table S1, the predicted target genes *Morus012124*, *Morus012122*, *Morus012121* of miRn51 also had the opposite expression pattern with miRn51. Four novel miRNA-target gene pairs (*Morus008520* for miRn247, *Morus019289* for miRn67, *Morus011908* for miRn62, *Morus002508* and *Morus014466* for miRn157) also possessed the opposite expression pattern with each other.

## Discussion

MiRNAs are important components in regulating plant physiological processes [1]. In the past several years, abundant conserved miRNAs and species-specific miRNAs were identified by high-throughput sequencing because of its high-throughput capacity in the detection of large-scale miRNAs and high sensitivity in the detection of minimally expressed miRNAs. In this study, sequencing of the three mulberry small RNA libraries was performed using Illumina technology. After analyzing millions of small RNA reads from these RNA libraries, 85 conserved miRNAs belonging to 31 families and 262 novel miRNAs at 371 loci were identified. After comparative analysis, several characteristics of conserved mulberry miRNAs were analyzed. A relationship between the degree of evolutionary conservation and the level of expression was observed in conserved miRNAs. As in previous studies, the majority of the conserved mulberry miRNA families identified here were evolutionarily conserved across plant species with high levels of expression. The less-conserved miRNA families (miR482, miR529, miR858, miR4376, miR4414, miR4995, and miR5523) showed lower abundance than the well-conserved miRNA families. The well- and less-conserved miRNA families may have evolved to play different roles in biological processes. The well-conserved mulberry miR164, miR167, miR156, miR172, miR159, miR166, miR171, miR172, and miR319 targeted NACs, ARFs, SPLs, APETALAs, MYBs, HD-ZIPIII, SCLs, AP2s, and TCPs, respectively. These transcription factors are very important to plant growth and development. For example, in *A. thalinana*, miR164 and miR167 affect lateral root development and adventitious rooting, respectively [37], [38]. miR167, miR159, miR160, and miR166 regulate the development of floral organs [39]–[46]. However, the targets of less-conserved miRNAs were mainly functional genes. It has recently been reported that miR482 and miR4376 target the NBS-LRR disease resistance gene and ACA10, respectively, and that they played a role in disease resistance and reproductive growth [47], [48]. Although well- and less-conserved miRNA families played different roles, both were found to be very important. Specifically, they cooperated to regulate the biological processes in plants.

Many minimally expressed and species-specific miRNA have been discovered in the plant kingdom using high-throughput sequencing. This indicated that each plant has its own specific miRNAs, which may play specific roles in physiological processes. In the present study, the characteristics of pre-miRNA including stem-loop structures and MFE, served as identification criteria for novel mulberry miRNAs. A total of 90 mulberry miRNA* and 262 novel mulberry miRNAs were identified. miRNA* is the product of dicer-like 1 (DCL1) and partial complementary to miRNA [49]. It was once considered to be degraded shortly after production and to have no roles in biological processes [50]. Recent studies have shown that miRNA* have important functions related to physiologically relevant levels, even though they are expressed at much lower levels than their miRNAs [51], [52]. It is speculated that the 90 low abundance miRNA* identified here might play important roles in various biological processes in mulberry plants. The novel mulberry miRNAs exhibited features different from those of conserved mulberry miRNAs. The expression levels of most novel mulberry miRNAs were very low, and they mainly targeted functional proteins. The predicted potential targets of mulberry novel miRNAs were involved in cellular processes, metabolic processes, response to stimulus, and metabolism. Research into possible targets in other plants may provide important clues to facilitate understanding of the function of these novel miRNAs. In the present study, one target of mno-miRn62 may be the disease resistance protein. This suggests that this novel miRNA may play a role in disease resistance in mulberry plants. In mulberries, flavanone 3-hydroxylase (F3H) has been found to participate in the anthocyanin biosynthesis pathway. The possible targets of four novel miRNAs (mno-miRn14, mno-miRn137, mno-miRn176, and mno-miRn252) encoded F3H and may play a role in regulating anthocyanin biosynthesis. Further study into novel mulberry miRNAs may shed light upon their roles in mulberry biological processes. This may fill in the blanks with respect to current knowledge of biological processes involving conserved miRNAs. Novel miRNAs that work with the conserved miRNAs might regulate plant development and response to the environment more broadly and accurately than either set of miRNA alone.

## Conclusions

This is the first comprehensive identification of conserved and novel miRNA in mulberry. The differential expression of miRNAs and the prediction of their target genes provide a basis for further understanding of mulberry miRNAs and the biological processes in which they are involved.

## Supporting Information

Figure S1 **Length distribution (A) and tissue distribution (B) of 85 conserved mulberry miRNAs.** Numbers in B indicate the number of conserved miRNAs.
(TIF)

Table S1 **The information of 262 novel mulberry miRNAs identified in three tissues.**
(XLSX)

Table S2 **Tissue-biased conserved miRNAs and their target genes.**
(XLSX)

Table S3 **Tissue-biased novel miRNAs and their target genes.**
(XLSX)

Table S4 **Tissue-specific novel mulberry miRNA.**
(XLSX)

Table S5 **Target genes of conserved mulberry miRNAs.**
(XLSX)

Table S6 **Target genes of novel mulberry miRNAs.**
(XLSX)

Table S7 **Primers used for stem-loop quantitative RT-PCR.**
(XLSX)

Table S8 **The primers of target genes used for quantitative RT-PCR.**
(XLSX)
