# Supplementary figures and images for: Identification of the Conserved and Novel miRNAs in Mulberry by High-Throughput Sequencing
Source: PLoS One. 2014 Aug 13;9(8):e104409. doi: 10.1371/journal.pone.0104409 (PMC4131894; doi:10.1371/journal.pone.0104409)

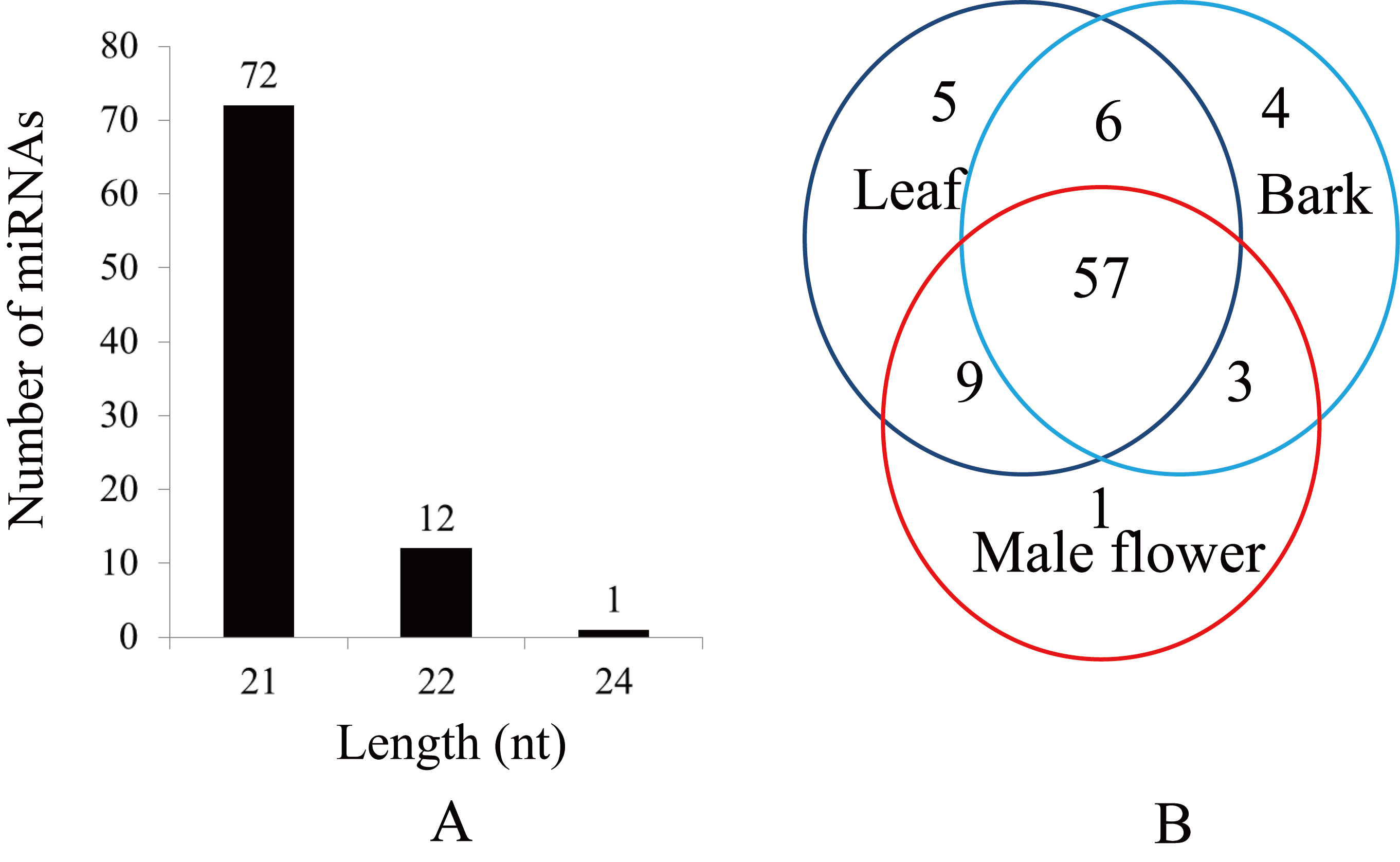

Supplement: Figure S1 — Length distribution (A) and tissue distribution (B) of 85 conserved mulberry miRNAs. Numbers in B indicate the number of conserved miRNAs. (TIF) [file pone.0104409.s001.tif]
